# Viral Etiology of Aseptic Meningitis and Clinical Prediction of Herpes Simplex Virus Type 2 Meningitis

**DOI:** 10.3390/jpm14090998

**Published:** 2024-09-20

**Authors:** Pamela Song, Jin Myoung Seok, Seungju Kim, Jaehyeok Choi, Jae Yeong Bae, Shi Nae Yu, Jongkyu Park, Kyomin Choi, Youngsoon Yang, Dushin Jeong, Kwang Ik Yang, Hyungkook Park

**Affiliations:** 1 Department of Neurology, Ilsan Paik Hospital, Inje University College of Medicine, Goyang 10380, Republic of Korea; 2 Department of Neurology, Soonchunhyang University Hospital Cheonan, Soonchunhyang University College of Medicine, 31 Soonchunhyang 6-gil, Dongnam-gu, Cheonan 31151, Republic of Korea; 3 Division of Infectious Diseases, Department of Internal Medicine, Soonchunhyang University Hospital Cheonan, Soonchunhyang University College of Medicine, Cheonan 31151, Republic of Korea

**Keywords:** aseptic meningitis, herpes simplex virus 2, machine learning, random forest, viral meningitis

## Abstract

Background: Aseptic meningitis comprises meningeal inflammation and cerebrospinal fluid (CSF) pleocytosis without positive Gram stain and culture. Regional differences exist in the prevalence of viral etiologies of aseptic meningitis. We aimed to assess the etiologies of aseptic meningitis in immunocompetent adults, focusing on herpes simplex virus type 2 (HSV-2). Methods: This study retrospectively analyzed immunocompetent adults diagnosed with meningitis at a Korean tertiary care hospital from 2016 to 2018. Aseptic meningitis was defined through clinical and CSF analysis. We compared clinical and laboratory characteristics across viral etiologies and investigated predictors of HSV-2 meningitis. Results: A total of 98 patients (46.9% female) with aseptic meningitis were finally enrolled. The etiologies of aseptic meningitis were identified in 62 patients (63.3%), including enterovirus (28.5%), HSV-2 (16.3%), and varicella zoster virus (VZV, 15.3%). HSV-2 showed female predominance, with shorter admission times with longer hospital stays and a recurrent meningitis history. Compared to other viral etiologies, HSV-2 showed higher CSF white blood cell (WBC) counts and protein levels but lower C-reactive protein (CRP) levels. A random forest model identified previous meningitis history and serum CRP level as key predictors of HSV-2 meningitis. Conclusions: This study provides insights into the etiologies of aseptic meningitis in a specific Korean region, identifying HSV-2 as a notable cause. The prediction model suggested that the clinical history of previous meningitis and serum CRP level may guide clinical assessment of meningitis.

## 1. Introduction

Aseptic meningitis is characterized by meningeal inflammation and cerebrospinal fluid (CSF) pleocytosis in the absence of a positive Gram stain or culture [1]. Identifying the etiology of meningitis is often clinically challenging; however, polymerase chain reaction (PCR) testing and more recent advances in multiplex PCR have greatly improved the ability to distinguish between bacterial, fungal, or aseptic meningitis [2,3]. While the viral etiology of most aseptic meningitis cases remains unknown, recognized viral etiologies are primarily enteroviruses, for which supportive care is needed for management [4,5]. On the other hand, antiviral agents have been recommended to treat other causes of viral meningitis, including varicella zoster virus (VZV) and herpes simplex virus (HSV) [5,6]. A few authors reported that more than half of the patients with aseptic meningitis could identify their viral etiologies using PCR testing on CSF samples [2,7,8]. However, aseptic meningitis could still be a management challenge in real clinical practice because of the under-use of diagnostic techniques for etiological analysis and the time required to identify the causative virus [9].

Herpes simplex virus type 2 (HSV-2) is an important and common cause of aseptic meningitis [10,11]. HSV-2 infection is one of the most prevalent sexually transmitted infections; the seroprevalence of HSV-2 varies significantly depending on age, sex, and geographic area [12,13,14]. Therefore, it is essential to analyze the occurrence of HSV-2 meningitis in specific geographic areas. However, there have been limited studies of HSV-2 meningitis in Korea; further, the clinical prediction of HSV-2 meningitis has never been studied before. In this study, we aimed to evaluate the etiologies of aseptic meningitis in immunocompetent adults and to investigate the clinical characteristics of HSV-2 meningitis.

## 2. Methods

### 2.1. Patients and Methods

We retrospectively reviewed the medical records of 143 patients who were diagnosed with meningitis and were admitted to the department of neurology at a tertiary care hospital in Korea between January 2016 and December 2018. Patients were enrolled if they had aseptic meningitis, which was defined as having acute symptoms and/or signs of meningeal inflammation, cerebrospinal fluid (CSF) pleocytosis (≥5/mm^3^), and the absence of microorganisms on Gram stain and/or culture [1]. Cases with encephalitis were excluded; encephalitis was defined according to the case definition used in previous studies that requires the presence of encephalopathy lasting longer than 24 h, along with one or more neurological signs and suggestive findings including fever, CSF pleocytosis, EEG abnormalities, or neuroimaging features [15]. All enrolled patients underwent full diagnostic workups for patients with meningitis, including routine laboratory blood tests, brain imaging (brain computed tomography (CT) or magnetic resonance imaging (MRI)), bacterial culture of blood and CSF, tests for tuberculosis, including polymerase chain reaction (PCR) for *Mycobacterium tuberculosis* and acid-fast bacilli (AFB) stain/culture, and microbiologic tests of CSF, including reverse transcription PCR test for enterovirus, and qualitative PCR tests for HSV type 1 (HSV-1), HSV-2, and VZV. The PCR test for the mumps virus was conducted for suspected patients who had pain and/or swollen salivary glands. Of the 143 cases initially reviewed, 45 were excluded from this study. These exclusions included one case of fungal meningitis, one case of HIV meningitis, eight cases of tuberculosis meningitis, eight cases of bacterial meningitis, and 27 cases due to incomplete diagnostic work-ups.

Data based on baseline characteristics, clinical features, laboratory findings, and brain images were assembled for patients with aseptic meningitis. This information included the following: age of onset; sex; time between symptom onset and admission; duration of hospital stay; history of previous meningitis; clinical symptoms profile including headache, fever, nausea/vomiting, neck stiffness, and cranial nerve palsy; blood test results of white blood cell count (WBC, /mm^3^); erythrocyte sedimentation rate (ESR, mm/h); C-reactive protein (CRP, mg/L) level; and CSF profiles including CSF WBC (/mm^3^), CSF protein level (mg/dL), and CSF-to-serum-glucose ratio. The local institutional review boards approved this study.

### 2.2. Statistical Analyses

Appropriate summary statistics were used to describe categorical and continuous variables. Continuous data were reported as mean ± standard deviation or as median with interquartile range (IQR). Categorical variables were presented in terms of absolute and relative frequencies. We analyzed the differences between groups according to the etiology of aseptic meningitis: VZV, HSV-2, enterovirus, and unknown cause. The Chi-square test or Fisher’s exact test were used for categorical variables, and continuous variables were analyzed using Student’s *t*-test, the Mann–Whitney U test, or the Kruskal–Wallis test. To predict the diagnosis of HSV-2 meningitis, we used random forest and logistic regression methods. The random forest algorithm is an ensemble supervised learning technique of multiple decision trees built using a bootstrap sample of the data. The random forest model was trained using all the clinical and laboratory variables from a randomly selected training set consisting of 70% of the enrolled patients; the remaining 30% were used as the test set. To assess the performance and general error of our random forest model, we used stratified 4-fold cross-validation and generated a receiver operating characteristic (ROC) curve with the area under the curve. A value of *p* < 0.05 was considered statistically significant. All statistical analyses were performed using SPSS for Windows version 20.0 (IBM, Armonk, NY, USA), and the ‘Scikit-Learn’ Python package (https://www.scikit-learn.org, accessed on 6 June 2023) was used for the machine learning model and prediction.

## 3. Results

### 3.1. Demographic Features and Etiology of Aseptic Meningitis

**Figure 1:**
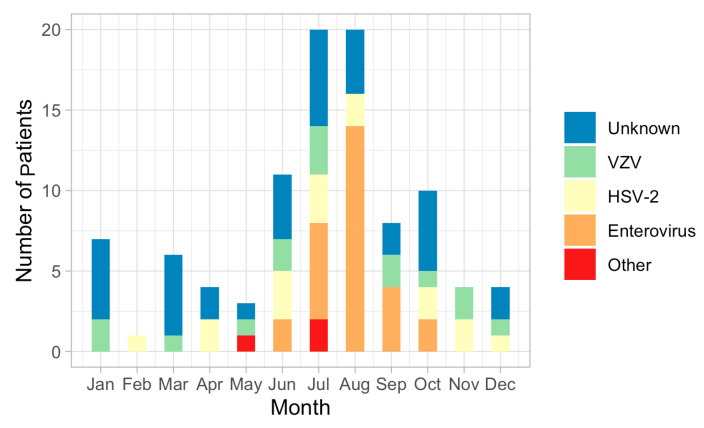
Etiologies of aseptic meningitis in immunocompetent adults and the change by month for a 2-year period.

A total of 98 patients with aseptic meningitis were finally enrolled; 46 (46.9%) patients were female, and 52 (53.1%) were male. The mean age of onset of aseptic meningitis was 33.0 ± 12.5 years. The number of cases of aseptic meningitis was high during the summer season: 20 patients with aseptic meningitis were observed in July and August, but only one in February. The etiology of aseptic meningitis was identified in 62 (63.3%) of the 98 enrolled patients but remained unknown in 36 patients (36.4%). The most common causative agent was enterovirus (28 patients, 28.5%), with a seasonal peak in cases during summer: enterovirus was identified in June, July, August, September, and October (*n* = 2, *n* = 6, *n* = 14, *n* = 4, and *n* = 2, respectively) (Figure 1). HSV-2 was the second most common etiology in patients with aseptic meningitis (16 patients, 16.3%); more than half of the patients with HSV-2 meningitis were female. Fifteen patients with aseptic meningitis were diagnosed with VZV. Detection patterns for both HSV-2 and VZV were similar throughout the year. HSV-1 meningitis was not observed. Three patients with Mycoplasma pneumoniae were identified.

### 3.2. Clinical and Laboratory Characteristics

The ages of onset among patients with HSV-2, VZV, and enterovirus were 29.7 ± 6.7 years, 40.9 ± 20.4 years, and 31.9 ± 4.9 years, respectively; the patients with HSV-2 tended to be younger than those with other etiologies. The median time between symptom onset and admission was 3.0 days (IQR 2.0–5.0 days), and the median duration of hospital was 5.0 days (IQR 4.0–8.0 days); these were significantly different among etiological groups (*p* < 0.001 and *p* = 0.040, respectively). In the HSV-2 meningitis group, the time between symptom onset and admission was shorter (median 2.0 days, IQR 1.3. 3.0 days), but they stayed longer in the hospital (median 6.5 days, IQR 4.0. 8.8 days) than those with other etiologies. Eleven patients were treated with intravenous acyclovir (10 mg/kg every eight hours) for median 7 days (range 4–10 days), six patients with VZV meningitis (6/15, 40%), four patients with HSV-2 meningitis (4/16, 25%), and one patient with meningitis of unknown cause (1/36, 2.8%). Among the groups, meningitis symptoms including headache, fever, nausea/vomiting, and neck stiffness were observed frequently, but there were no significant differences. Recurrent meningitis identified by history-taking was observed mostly in patients with HSV-2; 37.5% of HSV-2 patients had a previous history of meningitis. The clinical characteristics of patients according to etiology groups are presented in Table 1.

**Figure 2:**
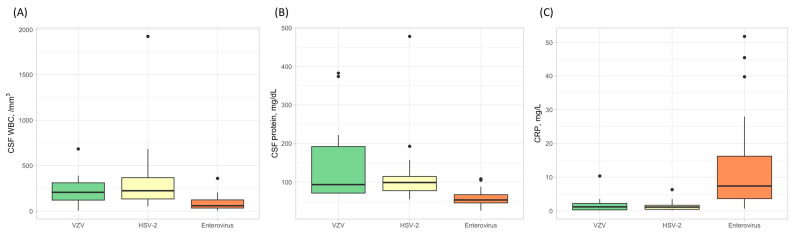
The white blood cell counts in cerebrospinal fluid (CSF), the level of CSF protein, and C-reactive protein (CRP) according to the etiologies in patients with aseptic meningitis. The CSF white blood cell counts were higher in patients with HSV-2 (**A**), while the CSF protein levels were elevated in the VZV group (**B**). In contrast, the levels of CRP were highest in the enterovirus group (**C**).

The results of the blood test and CSF study showed differences between etiology groups (Table 2). The level of CRP was significantly high (12.5 ± 13.5 mg/L, *p* < 0.001), but the WBC count of CSF was the lowest in patients with enterovirus meningitis (86.9 ± 82.3 /mm^3^, *p* < 0.001). The CSF protein level was the highest (150.8 ± 105.4 mg/dL, *p* < 0.001), but the CSF-to-serum-glucose ratio was the lowest (*p* < 0.001) in the VZV group. The patients with HSV-2 meningitis had elevated WBC counts and protein levels of CSF with low CRP levels compared to other etiology groups (Figure 2).

### 3.3. Prediction of HSV-2 Meningitis

The random forest model for classifying the diagnosis of HSV-2 meningitis had an accuracy of 0.900 (mean accuracy of 4-fold cross-validation models was 0.880), and the area under the ROC curve was 0.904 (95% CI, 0.826–0.981). The most important variables in descending order were the history of previous meningitis and CRP level (Supplementary Figures S1 and S2).

## 4. Discussion

In this study, we reported the viral etiologies of aseptic meningitis in adults and their clinical characteristics. The use of PCR in routine clinical practice would help in the rapid diagnosis and proper clinical decision-making among patients with meningitis [16]. Using PCR testing, the etiologies could be identified in more than 60% of enrolled patients, consistent with previous studies conducted in immunocompetent adults [7,8,17]. However, the prevalence of each etiology in this study was different from that in other reports. In our study, HSV-2 meningitis was the second most common cause of aseptic meningitis, but one Korean tertiary center study showed only one HSV-2 case among 177 patients with aseptic meningitis [7], and another study in the USA reported that HSV-2 meningitis was the most common etiology of aseptic meningitis in adults [9]. A UK study showed results consistent with ours, identifying HSV-2 meningitis as the second most common etiology in immunocompetent adults [11]. Notably, there was only one case of aseptic meningitis due to HIV infection; however, this case was excluded from our study due to the immunocompromised status. This observation may be attributed to the low prevalence of HIV infection in South Korea [18], as well as potential selection bias resulting from the enrollment of patients only from the neurology department.

The prevalence of HSV-2 infection is known to vary and depends on several factors. In general, HSV-2 prevalence is higher in women than in men and higher among commercial sex workers [12]; the group of HSV-2 meningitis cases in our study was younger than in other etiology groups, and more than half of the patients with HSV-2 were female. HSV-2 prevalence also varies markedly according to country or region in the country [12,14,19]. In this study, 16.3% of patients with aseptic meningitis had HSV-2 meningitis, but another study conducted in a different Korean city found that only 0.6% of the enrolled patients had HSV-2 meningitis [7]. Therefore, the diagnostic strategy of aseptic meningitis may differ according to region within the same country; therefore, regional seroprevalence data could be needed for proper clinical practice. Additionally, there were no cases of HSV-1 meningitis in our study. This finding is consistent with previous studies in Korea and may be attributed to our exclusion of encephalitis cases, as HSV-1 is more commonly associated with encephalitis [20].

Clinically presenting symptoms were similar regardless of the etiology, except for cranial nerve palsy and history of previous meningitis. Cranial nerve palsy was observed only among patients with VZV meningitis who were older than in those with different etiologies. VZV meningitis may be associated with neurologic complications; most patients with cranial nerve palsy had extraocular movement disturbance or facial palsy [21]. A history of previous meningitis was mostly observed in patients with HSV-2 meningitis (six patients with HSV-2 and three with unknown cause). The causes of recurrent aseptic meningitis are numerous, but among immunocompetent patients without structural lesions, recurrent aseptic meningitis is uncommon [22,23]. The syndrome of recurrent aseptic meningitis of unknown cause, previously called Mollaret’s meningitis, is now considered to be associated with HSV-2 meningitis [23,24]. Hence, it is conceivable that simple history taking about the previous meningitis may be an important clinical clue in the diagnosis of HSV-2 meningitis. The treatment strategy for HSV-2 meningitis is not well established; it is generally considered to be benign and self-limiting [6,25,26], and the use of intravenous acyclovir for patients with HSV-2 meningitis is highly variable [26,27]. In this study, the patients with HSV-2 meningitis were not treated with intravenous acyclovir, but they all recovered without sequelae. Further studies are needed to define the role of antiviral treatment in patients with HSV-2 meningitis. In addition to clinical characteristics, laboratory features could help diagnose HSV-2 infection. Patients with HSV-2 meningitis had high WBC counts in CSF without elevated CRP compared with patients of other etiologies; the level of CRP was significantly elevated in patients with enterovirus meningitis. Ihekwaba et al. also reported similar laboratory characteristics according to the etiology of aseptic meningitis, which could be helpful in distinguishing different etiologies of aseptic meningitis [16].

Machine learning has been studied across a variety of medical informatics applications to assist clinicians in improving their practice. It can serve various purposes, including facilitating diagnosis, predicting outcomes, risk stratification, and enhancing the quality of medical care [28,29]. In the field of infectious disease, machine learning is gaining trust as a new reliable technology. Luz C.F. et al. reported that logistic regression was the most commonly used algorithm, followed by random forest and support vector machines in this field. However, they also emphasized the need for independent model validation and clinical studies to evaluate the added value of machine learning approaches [30]. This study demonstrated the potential application of machine learning in patients with aseptic meningitis. It made use of the random forest algorithm, a popular tool for medical data analysis that has shown excellent performance in settings where the number of variables is larger than that of cases [31,32], and showed that the model could accurately predict the diagnosis of HSV-2 meningitis. Clinical factors, including history of previous meningitis and CRP level, were important factors in the predictive model, which suggested that basic clinical practices are valuable for diagnosis even in the absence of a PCR test. However, the interpretation of this result should be undertaken with caution since the etiologies of aseptic meningitis could vary greatly depending on geographic areas and characteristics of subpopulations. For the use of the random forest approach in clinical practice, further studies involving larger population samples with aseptic meningitis are needed.

This study has several limitations. This was a retrospective study that excluded patients with encephalitis and bacterial meningitis. Therefore, it is difficult to apply our study results to a real clinical setting. In addition, this study was conducted in a single tertiary center with a relatively small study population; therefore, data from different centers would be required to validate the prediction model. Moreover, we did not conduct a PCR test for all possible etiologies in aseptic meningitis, which can limit the application of our model.

In conclusion, this study offers insights into the etiologies of aseptic meningitis in a specific region of Korea, with HSV-2 identified as a notable cause, particularly in patients with a history of previous meningitis. While the developed machine learning model showed some promise as a tool for the clinical assessment of aseptic meningitis in this region, further studies with larger populations are necessary to refine the model and better understand its clinical applications.

## Figures and Tables

**Table 1:** Clinical characteristics of patients with aseptic meningitis according to etiology ^†^.

	Unknown Cause *n* = 36	VZV *n* = 15	HSV-2 *n* = 16	Enterovirus *n* = 28	*p*-Value ^‡^	*p*-Value *
Female, *n* (%)	15 (41.7)	6 (40.0)	10 (62.5)	13 (46.4)	0.524	0.422
Age, years (SD)	32.4 (13.9)	40.9 (20.4)	29.7 (6.7)	31.9 (4.9)	0.063	0.017
Time to admission, days (IQR)	4.5 (2.3–7.0)	4.0 (3.0–5.0)	2.0 (1.3–3.0)	2.0 (1.0–3.0)	<0.001	0.004
Duration of hospital stay, days (IQR)	5.0 (4.0–8.0)	6.0 (5.0–11.5)	6.5 (4.0–8.8)	4.5 (4.0–5.0)	0.040	0.014
Intravenous acyclovir treatment, *n* (%)	1 (2.8)	6 (40.0)	4 (25.0)	0 (0)		
Duration of acyclovir treatment, days (range)	7	7 (4–10)	7 (5–7)			
Cormobidities						
Hypertension, *n* (%)	2 (5.6)	1 (6.7)	0 (0)	3 (10.7)		
Diabetes mellitus, *n* (%)	2 (5.6)	1 (6.7)	0 (0)	0 (0)		
Previous history of meningitis, *n* (%)	3 (8.3)	0 (0)	6 (37.5)	0 (0)		
Presenting symtpoms						
Headache, *n* (%)	36 (100.0)	14 (93.3)	16 (100.0)	28 (100.0)	0.145	0.225
Fever, *n* (%)	28 (77.8)	11 (73.3)	15 (93.8)	23 (82.1)	0.469	0.313
Nausea or vomiting, *n* (%)	26 (72.2)	7 (46.7)	10 (62.5)	17 (60.7)	0.377	0.606
Neck stiffness, *n* (%)	18 (50.0)	8 (53.3)	11 (68.8)	15 (53.6)	0.655	0.573
Cranial nerve palsy, *n* (%)	0 (0)	2 (13.3)	0 (0)	0 (0)		

^†^ Four patients with aseptic meningitis by other causes were not presented in tables, as well as three patients with mycoplasma pneumoniae and one patient with HIV. ^‡^ *p*-value for all groups of aseptic meningitis. * *p*-value among three groups (VZV, HSV-2, and enterovirus group). VZV, varicella zoster virus; HSV-2, herpes simplex virus type 2; IQR, interquartile range. SD, stands for standard deviation.

**Table 2:** Laboratory findings of patients with aseptic meningitis according to etiology ^†^.

	Unknown Cause *n* = 36	VZV *n* = 15	HSV-2 *n* = 16	Enterovirus *n* = 28	*p*-Value ^‡^	*p*-Value *
WBC, /mm^3^ (SD)	8493 (3164)	7907 (2825)	8520 (2457)	8099 (2416)	0.892	0.784
ESR, mm/h (SD)	29.5 (19.9)	14.9 (9.8)	19.4 (15.1)	23.4 (12.2)	0.016	0.069
CRP, mg/L (SD)	15.0 (27.7)	1.9 (2.6)	1.5 (1.5)	12.5 (13.5)	<0.001	<0.001
CSF WBC, /mm^3^ (SD)	164.2 (184.7)	234.4 (168.7)	357.8 (453.0)	86.9 (82.3)	<0.001	<0.001
CSF protein, mg/dL (SD)	85.2 (47.1)	150.8 (105.4)	124.5 (100.3)	58.9 (19.5)	<0.001	<0.001
CSF-to-serum-glucose ratio, (SD)	0.54 (0.06)	0.48 (0.08)	0.50 (0.06)	0.55 (0.08)	<0.001	<0.001

^†^ Four patients with aseptic meningitis by other causes were not presented in tables, as well as three patients with mycoplasma pneumoniae and one patient with HIV. ^‡^ *p*-value for all groups of aseptic meningitis. * *p*-value among three groups (VZV, HSV-2, and enterovirus group). VZV, varicella zoster virus; HSV-2, herpes simplex virus type 2; WBC, white blood cells; ESR, erythrocyte sedimentation rate; CRP, C-reactive protein; CSF, cerebrospinal fluid; IQR, interquartile range.

## Data Availability

The datasets generated for this study are available upon request from the corresponding author.

## Supplementary Materials

The following supporting information can be downloaded at: https://www.mdpi.com/article/10.3390/jpm14090998/s1. Figure S1: Receiver operating characteristic curve for the random forest and logistic regression models; Figure S2: Importance of features.
