# Supplementary material for: Viral Etiology of Aseptic Meningitis and Clinical Prediction of Herpes Simplex Virus Type 2 Meningitis
Source: J Pers Med. 2024 Sep 20;14(9):998. doi: 10.3390/jpm14090998 (PMC11433182; doi:10.3390/jpm14090998)
Supplement: Supplementary file 1 [file jpm-14-00998-s001.zip › jpm-3170293-supplementary.pdf]

Supplementary Materials

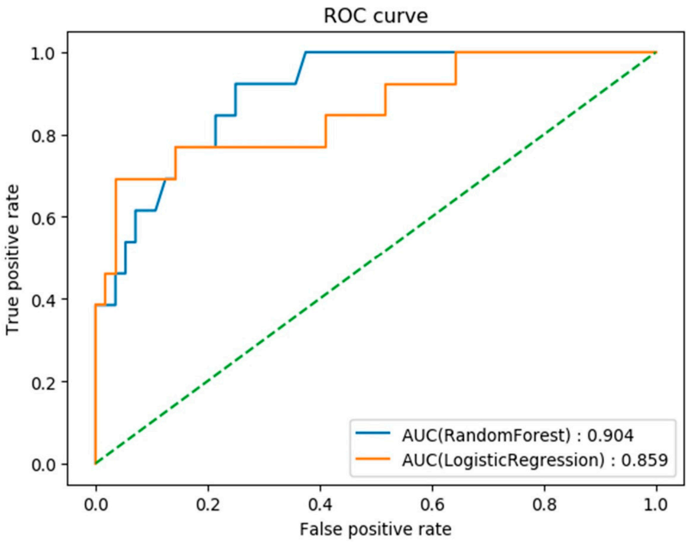

Figure S1. Receiver operating characteristic curve for the random forest and logistic regression models. The random forest model was superior with an area under the curve of 0.904 compared with the logistic regression model (0.859). ROC indicates receiver operating characteristic curve, and AUC, area under the curve.

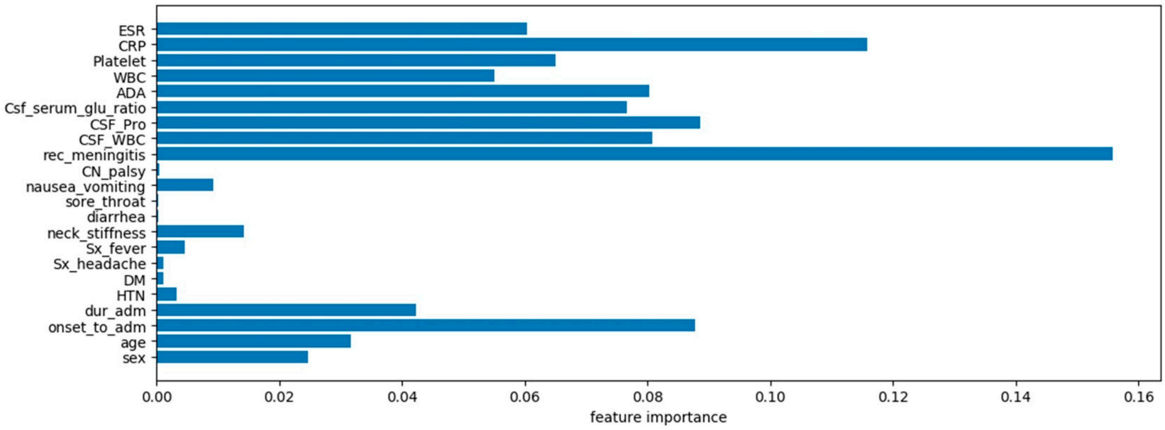

Figure S2. Importance of features.
